# Risk Factor Analysis for Developing Major Complications Following Esophageal Surgery—A Two-Center Study

**DOI:** 10.3390/jcm13041137

**Published:** 2024-02-17

**Authors:** Björn-Ole Stüben, Gabriel Andreas Plitzko, Louisa Stern, Rainer Schmeding, Karl-Frederick Karstens, Matthias Reeh, Jürgen Walter Treckmann, Jakob Robert Izbicki, Fuat Hakan Saner, Jan Peter Neuhaus, Michael Tachezy, Dieter Paul Hoyer

**Affiliations:** 1 Department of General-, Visceral- and Transplant Surgery, Medical Center University Duisburg-Essen, 45147 Essen, Germany; rainer.schmeding@uk-essen.de (R.S.); juergen-walter.treckmann@uk-essen.de (J.W.T.); fuat.saner@me.com (F.H.S.); jan.neuhaus@uk-essen.de (J.P.N.); dieter.hoyer@uk-essen.de (D.P.H.); 2 Department of General, Visceral and Thoracic Surgery, University Medical Center Hamburg-Eppendorf, 20246 Hamburg, Germany; gabriel@plitzko.net (G.A.P.); l.stern@uke.de (L.S.); kf.karstens@gmail.com (K.-F.K.); matthias.reeh@arcor.de (M.R.); izbicki@uke.de (J.R.I.); tachezym@gmail.com (M.T.)

**Keywords:** complications, esophagectomy, risk factors, risk analysis tool

## Abstract

Background: Esophagectomy carries a high risk of morbidity and mortality compared to other major surgeries. With the aim of creating an easy-to-use clinical preoperative risk assessment tool and to validate previously described risk factors for major complications following surgery, esophagectomies at two tertiary medical centers were analyzed. Methods: A total of 450 patients who underwent esophagectomy for esophageal carcinoma at the University Medical Centre, Hamburg, or at the Medical Center University Duisburg-Essen, Germany (January 2008 to January 2020) were retrospectively analyzed. Epidemiological and perioperative data were analyzed to identify the risk factors that impact major complication rates. The primary endpoint of this study was to determine the incidence of major complications. Results: The mean age of the patients was 63 years with a bimodal distribution. There was a male predominance across the cohort (81% vs. 19%, respectively). Alcohol abuse (*p* = 0.0341), chronic obstructive pulmonary disease (*p* = 0.0264), and cardiac comorbidity (*p* = 0.0367) were associated with a significantly higher risk of major complications in the multivariate analysis. Neoadjuvant chemotherapy significantly reduced the risk of major postoperative complications (*p* < 0.0001). Conclusions: Various patient-related risk factors increased the rate of major complications following esophagectomy. Patient-tailored prehabilitation programs before esophagectomy that focus on minimizing these risk factors may lead to better surgical outcomes and should be analyzed in further studies.

## 1. Introduction

The rising incidence of esophageal adenocarcinoma in developed countries [1], possibly due to significant increases in obesity rates and associated gastroesophageal reflux disease [2,3,4], is likely to lead to an increase in the number of esophagectomies in the coming years.

While local treatment options, such as endoscopic mucosal resection (EMR) and endoscopic submucosal dissection (ESD) [5] exist for early-stage malignancies, surgery remains the only curative option for most esophageal cancers. Open, hybrid, and total laparoscopic approaches exist as surgical options, and three common techniques for the resection of thoracic esophageal cancer exist, including the transhiatal approach, Ivor Lewis esophagectomy (laparotomy and right thoracotomy with intrathoracic anastomosis), and McKeown esophagectomy (laparotomy, right thoracotomy, and neck incision with cervical anastomosis) [6,7].

Although mortality after esophagectomy has decreased in the last 30 years, it is still associated with significant postoperative morbidity and relatively high in-hospital mortality rates of 7–9% [8]. The incidence of major postoperative complications ranges from 26 to 31%, with failure-to-rescue rates of 18–19% [9,10]. Some studies reported even higher complication rates, ranging from 17% to 74% [11,12]. The surgical technique does not appear to have a significant effect on postoperative complication rates, with a recent meta-analysis showing that laparoscopic and open approaches have similar outcomes regarding 5-year survival rates and postoperative complications, including anastomotic leakage, pulmonary infection, and arrhythmia [13].

Postoperative protocols, such as Enhanced Recovery for Surgery (ERAS), have been implemented with the aim of improving outcomes following major surgery [14].

Additionally, there is increased interest in improving the condition of patients prior to surgery. Identifying (modifiable) risk factors that increase the likelihood of major complications following surgery may improve patient outcomes by allowing clinicians to better prepare patients for surgery in individually conceptualized prehabilitation programs. Improving outcomes may also reduce the length of hospital stay (LOS), treatment costs, and readmission rates.

The goal of our study was to identify risk factors for postoperative complications, based on which clinicians seeing a patient in an ambulatory setting prior to esophagectomy can quickly and effectively assess the patient’s individual risk of developing postoperative complications.

## 2. Methods

### 2.1. Data Collection

All patients who underwent esophagectomy at the Department of General, Visceral, and Thoracic Surgery at the University Medical Centre Hamburg-Eppendorf or at the Department of General, Visceral, and Transplant Surgery at the Medical Center University Duisburg-Essen, Germany (admitted from January 2008 to January 2020) and were included in the prospective databases at each center were retrospectively analyzed. The patients underwent either totally minimally invasive, hybrid, or open esophagectomy. The epidemiological and perioperative data were analyzed to identify the risk factors that impact major complication rates.

The medical records of all participating patients, including their preoperative demographic data, comorbidities, and American Society of Anesthesiologists (ASA) score, were recorded.

Major complications were defined as Clavien-Dindo ≥ IIIa and only included complications mentioned in the consensus statement papers published by the Esophagectomy Complications Consensus Group (ECCG) for standardizing and benchmarking complications associated with esophagectomy [15,16]. With the aim of analyzing the risk factors that increased the rate of major complications, we investigated the effects of comorbidities and perioperative factors on major complication rates as the primary endpoint of the study.

In accordance with German law, approval by a local ethics committee was not required (paragraph 15, sentence 1, North Rhine Medical Association’s professional code of conduct from 14 November 1998, as amended on 19 November 2011), and written informed consent was not obtained from the participants because of the strict retrospective design of our study (paragraph 6, sentence 1, Health Data Protection Act of North Rhine-Westphalia).

### 2.2. Statistical Analysis

All statistical tests were performed using R Statistical software (version 4.2.1; R Foundation for Statistical Computing, Vienna, Austria) and GraphPad Prism software (version 9.2.0; GraphPad Software Inc., La Jolla, CA, USA). Quantitative variables with non-normal distributions are expressed as the median (interquartile range), and qualitative variables as sample sizes and percentages. Univariate associations between potential risk factors and major complications were assessed using the chi-square or Fisher’s exact test when the sample size was <5 for qualitative variables and the Mann–Whitney test for continuous variables with non-normal distributions. Multivariate analysis was performed using a logistic regression model to assess the predictors of major complications following esophageal surgery. All parameters with statistically significant relationships in the univariate analysis were introduced into the multivariate model to detect parameters independently associated with major postoperative complications. The Hosmer–Lemeshow test was used to evaluate the goodness of fit of the logistic model. The results are presented as odds ratios (ORs) with 95% CI. A receiver operating characteristic (ROC) curve was constructed, and the area under the ROC curve (AUC) was determined to assess the discriminant ability of the multiple regression model to predict major complications. The statistical significance level was set at *p* < 0.05, with all comparisons two-tailed.

## 3. Results

### 3.1. Baseline Demographics, Comorbidities

A total of 450 patients underwent esophagectomy for esophageal cancer at both centers between January 2008 and January 2020. Eleven patients were excluded from the analysis because of missing data. In total, 439 patients were included in the final statistical analysis. Of these, 329 were treated at the University Medical Center Hamburg-Eppendorf and 110 at the Medical Centre University Duisburg-Essen.

The median (interquartile range) patient age was 63 (56–71) years. There was a male predominance across the cohort (81% vs. 19%, respectively). Of these, 317 (72%) were treated for esophageal adenocarcinoma (EAC), while 116 (26%) were treated for squamous cell carcinoma (ESC). A total of 6 (1%) had other tumor entities. Among the analyzed patients, coronary heart disease (CHD) (13%), chronic obstructive pulmonary disease (COPD) (12%), diabetes mellitus (14%), and stroke (4%) were the most prevalent comorbidities. Smoking and regular alcohol consumption were found in 30% and 11% of the patients, respectively.

A total of 166 (38%) patients received neoadjuvant chemotherapy (CTx), and 91 (21%) patients received neoadjuvant radiochemotherapy.

Treatment data, major complications, LOS, and intensive care unit (ICU) stays were all included in the analysis.

A total of 95 (22%) patients underwent hybrid or totally minimally invasive esophagectomy, and 344 (78%) underwent open esophagectomy.

A total of 36 (8.2%) had Union for International Cancer Control (UICC) stage 0 disease, 128 (29%) had UICC 1, 78 (18%) had UICC 2, 162 (37%) had UICC 3, and 35 (8%) had UICC stage 4 disease.

Major complications included in the analysis were pneumonia, sepsis, pulmonary embolism (PE), postoperative bleeding, and anastomotic leakage. A total of 236 patients (54%) had at least one major complication.

The incidence of each major complication is shown in Table 1.

### 3.2. Univariate Analysis

We first analyzed each complication individually and the impact of perioperative risk factors on each complication. Owing to the small number of cases in each individual group and uneven distribution, the statistical power for each single complication was low. While neoadjuvant chemotherapy was significantly associated with lower complication rates for all complications, there were differences among other risk factors. Table 2 presents the results.

We subsequently analyzed the risk of developing ≥ 1 major complication and found that CHD (*p* = 0.041), COPD (*p* = 0.009), and alcohol consumption (*p* = 0.002) were associated with significantly higher major complication rates.

Neoadjuvant CTx was significantly associated with a lower rate of major complications (*p* < 0.001).

Importantly, the type of surgical procedure (minimally invasive, hybrid, or open) did not have a significant impact on major complication rates. Table 3 presents the results.

### 3.3. Outcomes

The median (interquartile range) LOS was 22 (15–36) days, and the median (interquartile range) ICU stay was 7 (4–17) days. Patients with major complications had a significantly longer median LOS (30 vs. 17 days, *p* < 0.001) and longer median ICU stay (13 vs. 4 days, *p* < 0.001).

### 3.4. Multivariate Analysis

With the aim of producing a simple risk score that could be easily applied in routine clinical practice, all parameters with statistically significant relationships in the univariate analysis were introduced into the multivariate model to detect parameters independently associated with major postoperative complications. The Hosmer–Lemeshow test was used to evaluate the goodness of fit of the logistic model.

This multivariate logistic regression model confirmed a statistically significant association between major complications following surgery for CHD (OR = 1.91, 95% CI 1.05–3.54, *p* = 0.0367), COPD (OR = 2.13, 95% CI 1.11–4.26, *p* = 0.0264), and alcohol consumption (OR = 2.16, 95% CI 1.09–4.56, *p* = 0.0341). Neoadjuvant CTx was protective against major complications (OR = 0.4, 95% CI 0.26–0.61, *p* < 0.0001). The cancer type did not have a statistically significant impact on complication rates.

These results are presented in Table 4.

### 3.5. Predictive Value of Comorbidities for Major Complications

We then created a clinical risk score to assess the patients according to their risk of developing complications. CHD, COPD, and alcohol consumption, which were all comorbidities associated with higher major complication rates, were each assigned an equal weight of one point, while neoadjuvant CTx was assigned a negative value, as it was shown to be associated with a lower risk of major complications.

**Figure 1:**
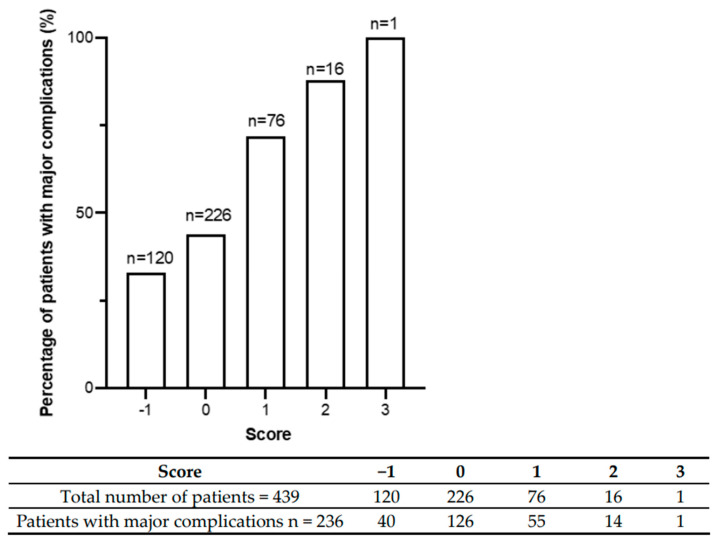
Major postoperative complication rates are shown according to cumulative risk score. The graph shows a monotonic increase in risk across increasing score values. Below the graph, the absolute number of patients distributed across the score categories is shown. Most patients had a score between −1 and 1.

The results are shown in Figure 1.

**Figure 2:**
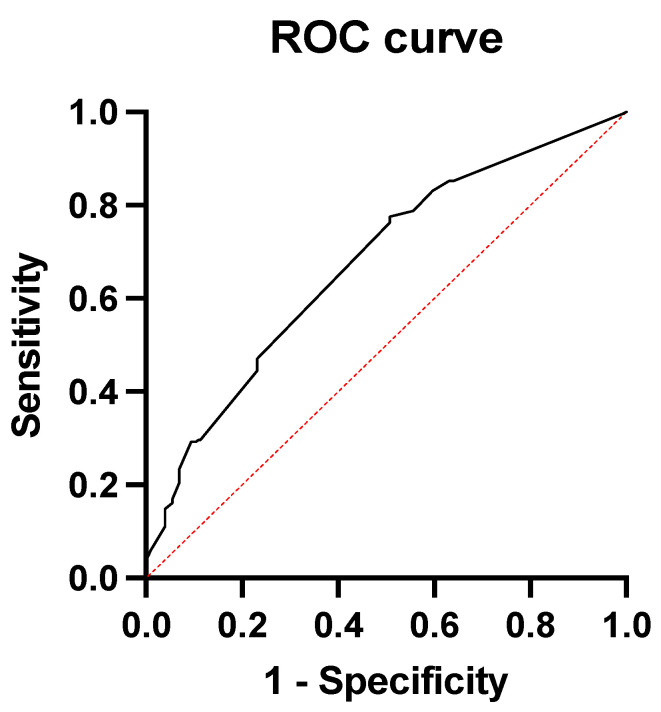
ROC curve showing the predictive value of the comorbidities for major complications following surgery.

An ROC curve was then constructed to visualize the trade-off between high sensitivity and high specificity in discriminating between low- and high-risk patients. The score based on comorbidities significantly correlated with the occurrence of major complications in patients following esophagectomy with an area under the curve (AUC) value of 0.675 (95% CI: 0.62–0.72, *p* < 0.0001). The ROC curve is shown in Figure 2.

## 4. Discussion

Esophagectomy is a surgical procedure with high rates of major complications and a relatively high in-hospital mortality rate. The identification of prognostic factors for adverse events after esophagectomy may provide opportunities to improve preoperative care by treating or optimizing these prognostic factors before surgery, thereby decreasing surgical risk.

Perioperative protocols, such as the implementation of the ERAS protocol in many medical centers, including the two tertiary medical centers involved in this analysis, have been shown to significantly shorten the length of hospital stay by 30% to 50%, with similar reductions in complications, while also reducing readmission rates and treatment costs [17]. Despite these positive results, some hospitals have been slow to adopt ERAS protocols, in part because of concerns regarding the expenses of program implementation. A cost calculation carried out by a group in the United States using data from existing publications and experience with the ERAS program at John Hopkins University calculated that the cost of USD 552,783 associated with implementation of an ERAS program was offset by even greater savings in the first year of nearly USD 948,500, yielding a net savings of USD 395,717 [18].

Another approach that has been receiving increased attention is the use of prehabilitation programs to optimize a patient’s condition prior to surgery.

Most patients undergo esophagectomy for esophageal malignancy. Primary resection is currently performed only in very early-stage cancers, with all others receiving multimodal therapy with either neoadjuvant chemotherapy or chemoradiation. This means that prehabilitation programs prior to surgery can be implemented, possibly in interdisciplinary cooperation with oncologists. Identifying modifiable risk factors that affect postoperative outcomes and increase major complication rates may help tailor these prehabilitation programs to the individual needs of patients. A systematic review showed that prehabilitation programs for patients with esophageal cancer can improve their fitness levels prior to surgery. However, the study failed to show significant effects on postoperative outcomes in these patients [19]. In addition to not having yet been shown to significantly improve outcomes in esophagectomy patients, prehabilitation programs are expensive due to the resources (medical personnel, physical activity programs, and dietary supplementation) that are required. To date, no cost analysis has been conducted for these prehabilitation programs before esophagectomy. If prehabilitation programs were to be shown to lead to similar improvements as ERAS protocols, the higher preoperative costs of these interventions could be offset by reduced postoperative costs as has been shown for ERAS protocols.

Several comorbidities have been shown to be correlated with major complication rates following esophagectomy. A meta-analysis from the Netherlands analyzed 39 studies with 48,853 patients undergoing esophagectomy. The authors identified 37 different patient-related prognostic factors for severe complications, anastomotic leakage, and/or 30-day/in-hospital mortality following esophagectomy [20]. A total of 23 prognostic factors for major postoperative complications (Clavien-Dindo ≥ IIIa) described in the analyzed studies were investigated. Male sex, diabetes, and respiratory and cardiac comorbidities were significantly associated with major complications.

In line with this, CHD and COPD were found to be significantly associated with postoperative complications in our study. We did not find any significant association with age. This may be due to the small age range (56–71) or the limited statistical power due to small cohort sizes.

Interestingly, the same meta-analysis showed lower anastomotic leakage rates after the administration of neoadjuvant therapy (CROSS or FLOT). The authors of the analysis suggested that this could have been due to a selection bias, as the administration of neoadjuvant therapy may have been omitted in frail patients unable to physically cope with neoadjuvant therapy; therefore, only patients with a better performance status received neoadjuvant therapy.

Like the above-mentioned meta-analysis, we are able to confirm that neoadjuvant CTx was significantly associated with lower anastomotic leakage rates. A novel finding in our study was that the risk of sepsis and pneumonia was also reduced, contributing to a lower overall major complication rate in patients who had received neoadjuvant therapy. While these results may appear surprising, they could be explained by a less metabolically active tumor disease in patients who show a good response to the therapy. Another explanation is that only patients with a relatively good performance status were deemed fit enough to receive chemotherapy prior to surgery, and possibly less radical surgery required after neoadjuvant therapy may have also contributed to lower complication rates. Whether these results are relatively unknown pathophysiological mechanisms or are due to selection bias in our study remains unclear, and it is possible that there are other risk or protective factors that we did not explore in this study that may have acted as confounders and contributed to the lower rate of postoperative complications.

Furthermore, we identified several risk factors associated with major complications after esophagectomy. In the multivariate analysis, we identified CHD, COPD, and alcohol consumption as factors that significantly increased the major complication rates.

The ROC analysis of our data showed that these risk factors accurately predicted the occurrence of major complications after surgery.

In creating a preoperative clinical risk assessment tool by incorporating risk factors significantly associated with major complications in the ROC analysis, we showed that there was a monotonic increase in the risk of major postoperative complications with increasing score values. Utilizing this clinical risk score could enable clinicians to identify patients who are vulnerable to major complications and to ensure that these patients entered a prehabilitation program prior to surgery to modify these individual risk factors. If proven in prospective studies, this may potentially lead to improved postoperative outcomes with shorter LOS and decreased healthcare system costs.

The present study had several major limitations. The small sample size, especially the small sample size of the different subgroups, certainly limits the statistical power of our data. The retrospective nature of the analysis further limits the strength of the results. In the univariate analysis, we statistically analyzed every complication on its own, with differences in sample sizes and relatively small case numbers for each complication, limiting the statistical power of the analysis and the ability of the model to predict the risk of each complication according to the risk factors. To increase the statistical power, we investigated the risk of developing at least one major complication.

The predictive power of our model is limited, and validation of the predictive power of our model with a separate, prospective independent patient cohort in a future study is necessary to validate the results of our analysis.

Our study cannot answer the question of whether prehabilitation programs do indeed improve postoperative results following esophagectomy, as standardized preoperative prehabilitation programs are not in place at either center involved in this study. Additionally, the ERAS guidelines for perioperative care in esophagectomy were published only in 2019 [21]. Prior to this, patients at our institutions were treated according to the ERAS guidelines for gastrectomy published in 2014 [22]. Patients treated before 2014 did not receive ERAS care. Subsequently, a heterogeneous cohort with differing perioperative protocols was compared in this study, which is a profound weakness.

Finally, due to the relatively restrictive implementation of minimally invasive surgical techniques at both institutions involved in this study, a large proportion of the patients in our study underwent open esophagectomy, which is no longer state-of-the-art. Currently, minimally invasive or hybrid esophagectomy is the standard of care at both centers. Whether our score is in fact applicable to minimally invasive procedures remains to be seen, and this should be addressed in future studies.

## 5. Conclusions

We identified various modifiable risk factors that increase the incidence of major complications associated with esophagectomy.

Our goal was to develop a score whose viability in a clinician’s daily routine is enhanced using parameters that can be easily and quickly identified in clinical practice to estimate the risk of postoperative complications. Therefore, a test with limited specificity can be used by clinicians to assess patients before surgery. In future studies, an independent prospective patient cohort is needed to validate the predictive power of our model and define a cut-off value for low- and high-risk patients.

## Figures and Tables

**Table 1:** Individual postoperative major complications.

Postoperative Complication	n (%)
Pneumonia	160 (36%)
Sepsis	94 (21%)
PE	26 (5.9%)
Bleeding	13 (3.0%)
Anastomotic leakage	111 (25%)
≥1 Major Complication	236 (54%)

PE: pulmonary embolism.

**Table 2:** Univariate analysis of demographic and clinical data for each major complication.

**Variable**	**Anastomotic Insufficiency**			**Surgical Site Infection**			**Bleeding**		
	**No**	**Yes**		**No**	**Yes**		**No**	**Yes**	
	**n = 328**	**n = 112**	***p*** **-Value**	**n = 404**	**n = 35**	***p*** **-Value**	**n = 426**	**n = 13**	***p*** **-Value**
**Age (years)**	63 (56, 71)	62 (57, 70)	0.8	63 (56, 71)	60 (54, 70)	0.6	63 (56, 71)	59 (57, 64)	0.5
**Male sex**	266 (81%)	92 (82%)	0.8	326 (81%)	31 (89%)	0.3	345 (81%)	12 (92%)	0.5
**Diabetes**	50 (15%)	11 (9.9%)	0.2	57 (14%)	4 (11%)	0.8	59 (13%)	2 (15%)	0.7
**CHD**	44 (13%)	15 (14%)	>0.9	57 (14%)	2 (5.7%)	0.2	55 (13%)	4 (31%)	0.083
**Stroke**	17 (5.2%)	2 (1.8%)	0.2	17 (4.2%)	2 (5.7%)	0.7	19 (4.5%)	0 (0%)	>0.9
**COPD**	37 (11%)	14 (13%)	0.7	49 (12%)	2 (5.7%)	0.4	50 (12%)	1 (7.7%)	>0.9
**Alcohol**	25 (7.6%)	22 (20%)	**<0.001**	42 (10%)	5 (14%)	0.4	43 (10%)	4 (31%)	**0.040**
**Tobacco use**	88 (27%)	43 (39%)	**0.018**	117 (29%)	14 (40%)	0.2	125 (29%)	6 (46%)	0.2
**CTx**	142 (43%)	24 (21%)	**<0.001**	153 (38%)	13 (37%)	>0.9	164 (38%)	2 (15%)	0.14
**Open surgery**	260 (79%)	85 (76%)	0.5	312 (77%)	32 (91%)	0.05	335 (79%)	9 (69%)	0.5
**UICC stage**			0.6			0.3			0.9
0	25 (8%)	11 (10%)		35 (8.7%)	1 (2.9%)		34 (8.0%)	2 (15%)	
1	99 (30%)	29 (26%)		121 (30%)	7 (20%)		125 (29%)	3 (23%)	
2	53 (16%)	25 (22%)		71 (18%)	7 (20%)		76 (18%)	2 (15%)	
3	128 (39%)	35 (31%)		147 (36%)	15 (43%)		157 (37%)	5 (38%)	
4	23 (7%)	12 (11%)		30 (7.4%)	5 (14%)		34 (8.0%)	1 (7.7%)	
**Type of Cancer**			**<0.001**			0.3			0.5
EAC	251 (77%)	67 (60%)		290 (72%)	27 (77%)		309 (73%)	8 (62%)	
ESC	71 (22%)	45 (40%)		109 (27%)	7 (20%)		111 (26%)	5 (38%)	
Other	6 (1.8%)	0 (0%)		5 (1.2%)	1 (2.9%)		6 (1.4%)	0 (0%)	
**Variable**	**Pneumonia**			**Sepsis**			**PE**		
	**No**	**Yes**		**No**	**Yes**		**No**	**Yes**	
	**n = 279**	**n = 160**	***p*** **-Value**	**n = 345**	**n = 95**	***p*** **-Value**	**n = 413**	**n = 26**	***p*** **-Value**
**Age (years)**	63 (56, 71)	64 (57, 71)	0.7	63 (56, 71)	64 (57, 71)	0.5	63 (56, 71)	58 (56, 64)	0.11
**Male sex**	223 (80%)	134 (84%)	0.3	281 (81%)	77 (81%)	>0.9	335 (81%)	22 (85%)	0.8
**Diabetes**	40 (14%)	21 (13%)	0.7	48 (14%)	13 (14%)	>0.9	58 (14%)	3 (12%)	>0.9
**CHD**	35 (13%)	24 (15%)	0.5	38 (11%)	21 (22%)	**0.004**	55 (13%)	4 (15%)	0.8
**Stroke**	15 (5.4%)	4 (2.5%)	0.2	16 (4.6%)	3 (3.2%)	0.8	19 (4.6%)	0 (0%)	0.6
**COPD**	23 (8.2%)	28 (18%)	**0.004**	38 (11%)	13 (14%)	0.5	50 (12%)	1 (3.8%)	0.3
**Alcohol**	22 (7.9%)	25 (16%)	**0.012**	32 (9.3%)	15 (16%)	0.063	46 (11%)	1 (3.8%)	0.3
**Tobacco use**	78 (28%)	53 (33%)	0.3	101 (29%)	30 (32%)	0.6	124 (30%)	7 (27%)	0.7
**CTx**	126 (45%)	40 (25%)	**<0.001**	145 (42%)	22 (23%)	**<0.001**	155 (38%)	11 (42%)	0.6
**Open surgery**	216 (77%)	132 (82%)	0.2	265 (77%)	80 (84%)	0.12	324 (78%)	20 (77%)	0.9
**UICC stage**			0.4			0.6			**0.015**
0	29 (10%)	7 (4.4%)		30 (8.7%)	7 (7.4%)		33 (8.0%)	3 (12%)	
1	80 (29%)	48 (30%)		102 (30%)	26 (27%)		121 (29%)	7 (27%)	
2	48 (17%)	30 (18%)		64 (19%)	14 (15%)		78 (19%)	0 (0%)	
3	98 (35%)	64 (40%)		122 (35%)	40 (42%)		151 (37%)	11 (42%)	
4	24 (9%)	11 (6.9%)		27 (7.8%)	8 (8.4%)		30 (7.3%)	5 (19%)	
**Type of Cancer**			0.4			0.3			
EAC	206 (74%)	111 (69%)		252 (73%)	65 (68%)		295 (71%)	22 (85%)	0.5
ESC	68 (24%)	48 (30%)		87 (25%)	30 (32%)		112 (27%)	4 (15%)	
Other	5 (1.8%)	1 (0.6%)		6 (1.7%)	0 (0%)		6 (1.5%)	0 (0%)	

CHD: coronary heart disease, COPD: chronic obstructive pulmonary disease, CTx: chemotherapy, UICC: Union for International Cancer Control. EAC: esophageal adenocarcinoma, ESC: esophageal squamous cell carcinoma. Data are presented as median (interquartile range) or n (%). Statistical significance was tested using the chi-square or Fisher’s exact test when the sample size was <5 for qualitative variables and with the Mann–Whitney test for continuous variables with non-normal distributions. Bold *p*-values are of statistical significance (*p* ≤ 0.05).

**Table 3:** Univariate analysis for the development of at least one major complication.

Variable	Total	≥1 Major Complication		
		No	Yes	
	n = 441	n = 203	n = 238	*p*-Value
**Age (years)**	63 (56, 71)	63 (56, 71)	63 (57, 71)	>0.9
**Male sex**	359 (81%)	161 (79%)	198 (83%)	0.3
**Diabetes**	61 (14%)	31 (15%)	30 (13%)	0.4
**CHD**	59 (13%)	20 (9.9%)	39 (17%)	**0.041**
**Stroke**	19 (4%)	11 (5.4%)	8 (3.4%)	0.3
**COPD**	51 (12%)	15 (7.4%)	36 (15%)	**0.010**
**Alcohol**	47 (11%)	12 (5.9%)	35 (15%)	**0.003**
**Tobacco use**	131 (30%)	57 (28%)	74 (31%)	0.5
**CTx**	167 (38%)	102 (50%)	65 (27%)	**<0.001**
**Open surgery**	346 (78%)	161 (79%)	185 (78%)	0.700
**UICC stage**				0.6
0	37 (8%)	20 (9.9%)	17 (7.1%)	
1	128 (29%)	62 (31%)	66 (28%)	
2	78 (18%)	35 (17%)	43 (18%)	
3	163 (37%)	68 (33%)	95 (40%)	
4	35 (8%)	18 (8.9%)	17 (7.1%)	
**Type of Cancer**				**0.005**
EAC	318 (72%)	159 (78%)	159 (67%)	
ESC	117 (27%)	40 (20%)	77 (32%)	
Other	6 (1.4%)	4 (2.0%)	2 (0.8%)	

CHD, coronary heart disease; COPD, chronic obstructive pulmonary disease; CTx, chemotherapy; UICC, Union for International Cancer Control. EAC, esophageal adenocarcinoma; ESC, esophageal squamous cell carcinoma. Data are presented as median (interquartile range) or n (%). Statistical significance was tested using the chi-square or Fisher’s exact test when the sample size was <5 for qualitative variables and with the Mann–Whitney test for continuous variables with non-normal distributions. Bold *p*-values are statistically significant (*p* ≤0.05).

**Table 4:** Multivariate logistic regression analysis of preoperative risk factors associated with major complications.

Variable	Odds Ratios (CI 95%)	*p* Value
CHD	1.91 (1.05–3.54)	**0.0367**
COPD	2.13 (1.11–4.26)	**0.0264**
Alcohol	2.16 (1.09–4.56)	**0.0341**
CTx	0.40 (0.26–0.61)	**<0.0001**
Type of Cancer	1.39 (0.86–2.25)	0.1786

CHD, coronary heart disease; COPD, chronic obstructive pulmonary disease; CTx, chemotherapy. Bold *p*-values, statistical significance (≤0.05). Marked red: associated with a decreased risk of major complications.

## Data Availability

The data supporting the findings of the study are available upon request from the corresponding author (B.-O.S.).
